# Selective-Area Growth of Transferable InN Nanocolumns by Using Anodic Aluminum Oxide Nanotemplates

**DOI:** 10.1186/s11671-017-1924-0

**Published:** 2017-02-23

**Authors:** Xiao Wang, Guozhen Zhang, Yang Xu, Hao Wu, Chang Liu

**Affiliations:** Key Laboratory of Artificial Micro- and Nano-structures of Ministry of Education, School of Physics and Technology, Wuhan University, Wuhan, 430072 People’s Republic of China

**Keywords:** InN nanocolumns, Anodic aluminum oxide, Selective-area growth

## Abstract

InN nanocolumn arrays were grown on c-plane sapphire with and without anodic aluminum oxide (AAO) nanotemplates. The crystalline quality of InN nanocolumns was significantly improved by selective-area growth (SAG) using AAO templates, as verified by X-ray diffraction measurements. Then, InN nanocolumns were transferred onto p-type silicon substrates after etching off the AAO templates. Current–voltage characteristic of the transferred n-InN/p-Si heterojunctions shows on/off ratio as high as 4.65 × 10^3^ at 2 V. This work offers a potential way to grow transferable devices with improving performances.

## Background

III-Nitrides, with excellent optic and electronic properties, are widely used for solar cells, optical waveguides, high-speed electronics, and terahertz emitters [1]. Among them, InN has the narrowest bandgap, lowest effective mass of electrons, and highest electron mobility and thus can be applied in high-speed electronics [2–4]. However, its low decomposition temperature, impurity-prone surface, and large lattice mismatch with common substrates hinder the further development of InN-based devices [1]. In recent years, many studies focused on the growth of InN nanorods and nanocolumns on c-Al_2_O_3_, glass, Si (100), and Si (111) with or without buffer layers such as GaN, AlN, InGaN, and even low-temperature (LT) InN [5–10]. In general, sapphire is widely used as the substrate for growth of InN because of its availability, large area, high quality, high thermal stability, relatively low cost, and hexagonal symmetry, even though it has a large lattice mismatch with InN [11].

Selective-area growth (SAG) technique has been used to produce waveguides [12], facet lasers [13], and other nanostructures such as nanowires and nanocolumns [14–16]. It has been applied to achieve the epitaxial lateral overgrowth (ELOG) of GaN-based laser diodes for reducing the threading dislocation density. Until now, there are only a few reports on the SAG of InN, in which Mo-mask-patterned (0001) sapphire [11], Mo-mask-patterned (111) Si [17], nanohole-patterned GaN templates [18], and ultra-thin AlN masks were used [19]. However, the AlN masks are not periodical, and the fabrication of the metal masks is relatively expensive and complex. Hence, more periodical and practical nano-sized masks should be developed for the SAG of InN-based devices.

Anodic aluminum oxide (AAO) templates are widely applied in nanostructure fabrication due to their high regularity, self-organized nanostructure, and low cost compared with electron beam lithography (EBL) [20]. Furthermore, AAO is more easily etched compared to Pt, Mo, and AlN. Therefore, AAO templates were used in this study as both the SAG mask and the transfer template.

## Methods

**Fig. 1:**
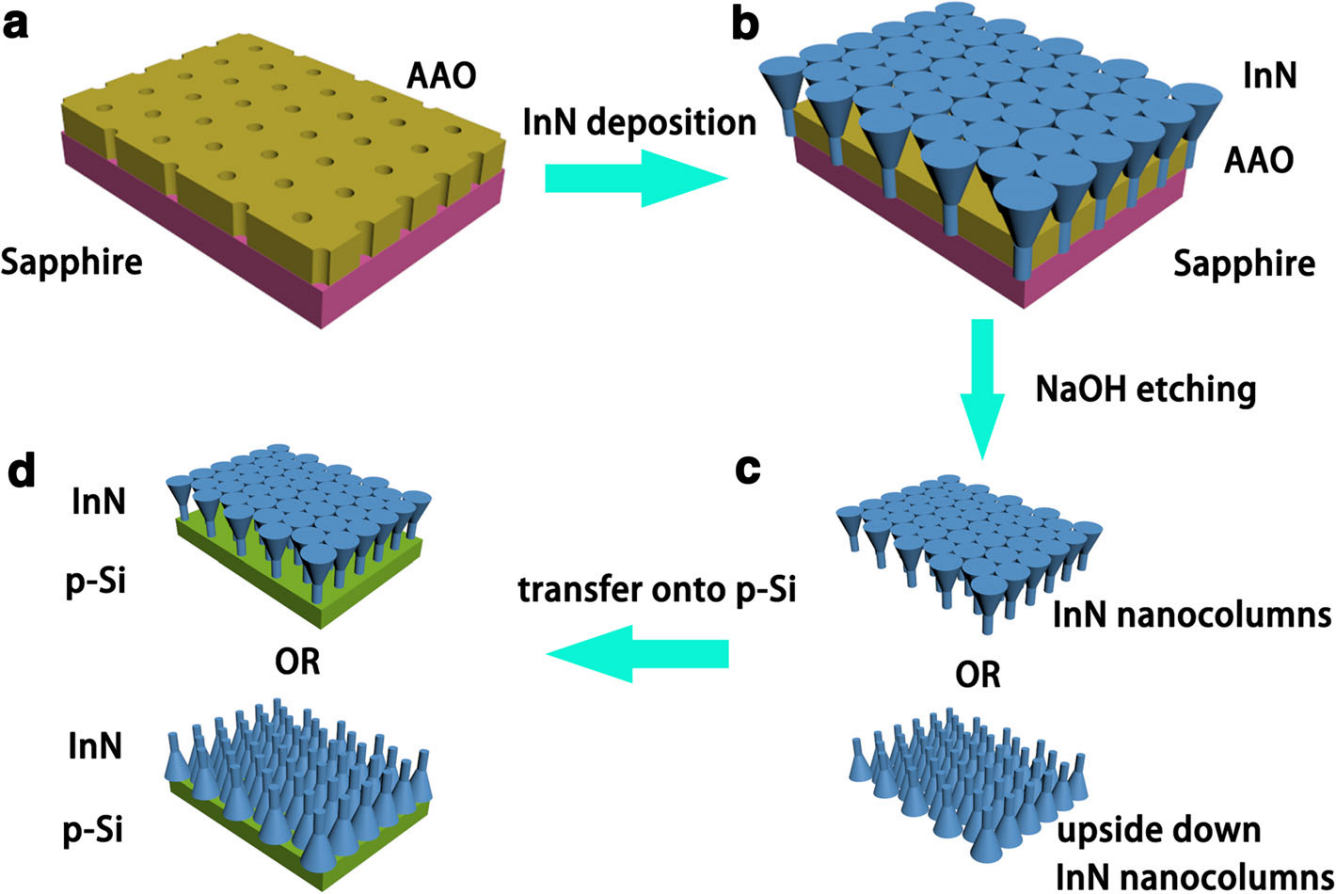
Schematic diagram of the fabrication process. **a** AAO templates on sapphire substrate. **b** InN nanocolumns deposited on AAO templates. **c** Free-standing InN nanocolumns. **d** Transferred InN nanocolumns on p-Si

**Fig. 2:**
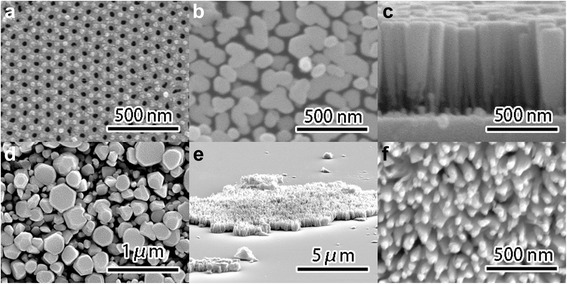
Cross-sectional SEM images of **a** AAO templates, **b** top view of InN nanocolumns without the AAO templates, **c** side view of InN nanocolumns without the AAO templates, **d** top view of InN nanocolumns with the AAO templates, **e** transferred upside down InN nanocolumns on p-Si, and **f** top view of the transferred upside down InN nanocolumns

Figure 1 shows the schematic diagram of the fabrication process: High-purity (99.999%) aluminum foils were used to fabricate the AAO templates through a typical two-step anodizing procedure. According to the anodization conditions, the pore diameter and interpore edge-to-edge distance were expected to be 30 and 70 nm, which was shown in Fig. 2a. The depth of AAO pores was 170 nm. After anodization, the aluminum base and barrier layers were removed completely out and the free-standing AAO templates were transferred onto sapphire substrates. Details of anodization and transferring methods can be found elsewhere [21]. The sapphire wafers used in this work were ultrasonically cleaned in acetone for 10 min and rinsed with ethanol for 10 min to remove organic grease. InN nanocolumns were then grown on sapphire substrates with and without AAO templates for 2 h by radio-frequency molecular beam epitaxy (RF-MBE, SVTA 35-V-2). The temperatures of In source and the substrate were set at the optima 770 and 400 °C, respectively [10]. Prior to the growth, nitridation was performed under 500 W RF-plasma power at 500 °C for 10 min in a nitrogen atmosphere with a flow rate of 2.65 sccm. The InN nanocolumns were characterized by high-resolution X-ray diffraction (HRXRD, Bede D1) and scanning electron microscopy (SEM, FEI, Versa 3D). The as-grown samples were then immerged into NaOH solution with a concentration of 10% at 50 °C for 10 min to remove the AAO templates and then rinsed with deionized water to remove the NaOH solution and reaction products. After NaOH solution etching, the detached InN nanocolumns randomly floated in the deionized water. The InN nanocolumns were subsequently transferred onto p-Si substrates either in growth direction or upside down. Current versus voltage (*I*–*V*) characteristics of the transferred n-InN/p-Si heterojunctions were measured by using a semiconductor device analyzer (Keithley 4200, Keithley Instruments).

## Results and Discussion

Figure 2 shows the cross-sectional SEM images. The AAO templates were found with an average pore diameter of 30 nm and interpore edge-to-edge distance of 70 nm as well as pore depth of 170 nm, as shown in Fig. 2a. The density of the pores was estimated to be 1.2 × 10^10^ cm^−2^. As shown in Fig. 2b, the diameters of the InN nanocolumns without AAO templates varied from 50 to 150 nm in top view. The density of the InN nanocolumns without the AAO templates was estimated to be 8 × 10^9^ cm^−2^. As shown in Fig. 2c, the depth of the nanocolumns was 625 nm. As shown in Fig. 2d, the diameters of the InN nanocolumns with the AAO templates varied from 50 to 500 nm. The density of the InN nanocolumns with the AAO templates was estimated to be 1 × 10^9^ cm^−2^. The decrease of the InN nanocolumns densities was mainly attributed to the selective lateral growth by adding the AAO templates. As shown in Fig. 2e, the InN nanocolumns can be either in growth direction or upside down after being transferred onto p-Si by etching AAO templates off using NaOH solution. The sizes of the transferred InN nanocolumns were in micrometer scales from 2 × 2 to 20 × 20 μm^2^. Fig. 2f shows the top view of the transferred upside down InN nanocolumns. The density of the transferred upside down InN nanocolumns with the AAO templates was estimated to be 1.1 × 10^10^ cm^−2^, which was in agreement with the density of the AAO template pores as shown in Fig. 2a. This indicates that the InN nanocolumns were selective-area grown from the pores of the AAO templates.

**Fig. 3:**
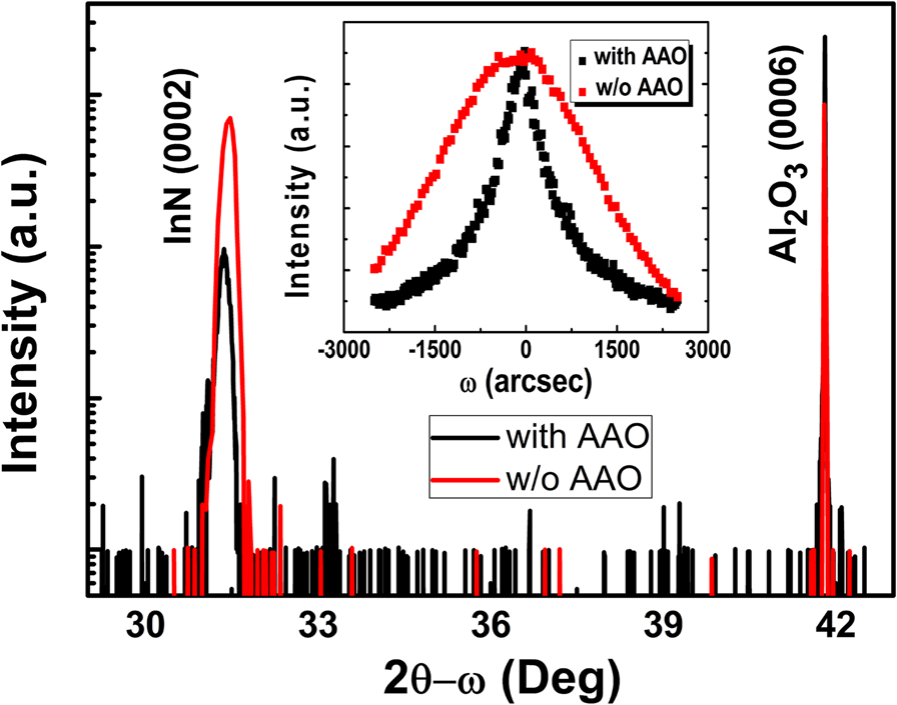
XRD spectra of the InN nanocolumns with and without the AAO templates. The *inset* shows the rocking curves around InN (0002)

Figure 3 shows X-ray diffraction (XRD) patterns of the InN nanocolumns with and without the AAO templates. Two peaks were detected in 2θ-ω scan. The peaks around 31.4° and 41.9° were attributed to InN (0002) and sapphire (0006), respectively. It can be clearly seen that the InN (0002) peak with the AAO templates is much sharper compared to that without the AAO templates. This indicates that the crystalline quality of the InN nanocolumns is improved by using the SAG with the AAO templates. Furthermore, the InN (0002) peak with AAO templates shifts to smaller 2 θ region, approaching the native value of the free-standing InN films at 31.3°. The inset shows the rocking curves of InN (0002) with and without AAO templates after normalization. The full widths at half maximum (FWHM) of InN (0002) with and without the AAO templates are 1260″ and 2988″ after fitting with Gaussian curve, respectively, indicating a significantly improved crystalline quality of InN nanocolumns by the SAG using AAO templates.

**Fig. 4:**
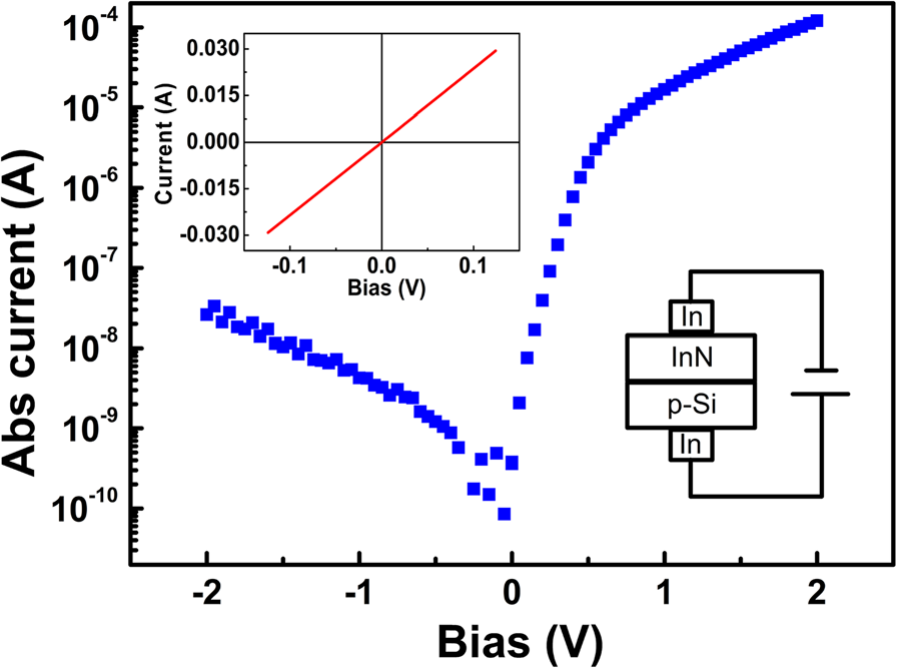
*I*–*V* characteristics of transferred n-InN/p-Si heterojunction. The *inset* shows the sketch map of configuration and ohmic contact of the indium electrodes

Figure 4 shows the current–voltage (*I*–*V*) characteristic of the transferred n-InN/p-Si heterojunctions. The indium electrodes were welded on the surfaces of the transferred InN and Si layers to achieve ohmic contacts. The inset shows the ohmic contact between the indium electrodes and p-Si. *I*–*V* curves were measured by changing the bias from +2 to −2 V. The currents were restricted between 30 and −30 mA to protect the devices. Diode-like behaviors were obtained. Under reverse bias of −2 V, the leakage current of the transferred n-InN/p-Si heterojunctions was about 26 nA, while the current under forward bias of 2 V was about 121 mA. On/off ratio (current ratio *I*_pos_/*I*_neg_ under the positive voltage (+2 V) and negative voltage (−2 V)) was as high as 4.65 × 10^3^, which was much higher than the value reported previously [22]. This high on/off ratio provides more potential for the application of n-InN/p-Si diodes. Under the forward biases, the turn-on voltage of the transferred n-InN/p-Si heterojunctions was fitted to be 0.67 V, which was comparable to the average barrier height of 0.7 V in Bhat’s work [22]. Thus, the excellence of the transferred InN nanocolumns and the n-InN/p-Si heterojunctions can be deduced.

## Conclusions

In this study, InN nanocolumn arrays were grown on c-plane sapphire with and without the AAO nanotemplates. The crystalline quality of the InN nanocolumns is significantly improved by the SAG using the AAO nanotemplates, as verified by XRD measurements. *I*–*V* characteristic of the transferred n-InN/p-Si heterojunctions shows on/off ratio as high as 4.65 × 10^3^ at 2 V. This work offers a potential way to grow high-quality transferable devices with improving performances.
